# OsMYC2, an essential factor for JA-inductive sakuranetin production in rice, interacts with MYC2-like proteins that enhance its transactivation ability

**DOI:** 10.1038/srep40175

**Published:** 2017-01-09

**Authors:** Satoshi Ogawa, Koji Miyamoto, Keiichirou Nemoto, Tatsuya Sawasaki, Hisakazu Yamane, Hideaki Nojiri, Kazunori Okada

**Affiliations:** 1 Biotechnology Research Center, The University of Tokyo, Bunkyo-ku, Tokyo, Japan; 2 Department of Biosciences, Teikyo University, Utsunomiya, Tochigi, Japan; 3 Proteo-Science Center, Ehime University, Matsuyama, Ehime, Japan

## Abstract

Biosynthesis of sakuranetin, a flavonoid anti-fungal phytoalexin that occurs in rice, is highly dependent on jasmonic acid (JA) signalling and induced by a variety of environmental stimuli. We previously identified *OsNOMT*, which encodes naringenin 7-*O*-methyltransferase (NOMT); NOMT is a key enzyme for sakuranetin production. Although *OsNOMT* expression is induced by JA treatment, the regulation mechanism that activates the biosynthetic pathway of sakuranetin has not yet been elucidated. In this study, we show that JA-inducible basic helix-loop-helix transcriptional factor OsMYC2 drastically enhances the activity of the *OsNOMT* promoter and is essential for JA-inducible sakuranetin production. In addition, we identified 2 collaborators of OsMYC2, OsMYC2-like protein 1 and 2 (OsMYL1 and OsMYL2) that further activated the *OsNOMT* promoter in synergy with OsMYC2. Physical interaction of OsMYC2 with OsMYL1 and OsMYL2 further supported the idea that these interactions lead to the enhancement of the transactivation activity of OsMYC2. Our results indicate that JA signalling via OsMYC2 is reinforced by OsMYL1 and OsMYL2, resulting in the inductive production of sakuranetin during defence responses in rice.

Jasmonic acid (JA) and its derivatives, the so called jasmonates, have been extensively characterized as the central players during biotic stress resistance, for example, emission of volatiles to attract the natural enemies of herbivores or production of toxic compounds to repel invaders^1^,^2^,^3^,^4^. In rice, jasmonates induce the production of anti-microbial specialized metabolites called phytoalexins, which consist of 16 diterpenoids and 1 flavonoid^5^,^6^. Among them, the flavonoid sakuranetin is considered to be the biologically important phytoalexin in terms of its high accumulation in blast-infected rice leaves and high anti-fungal activity^7^,^8^. Furthermore, its biosynthesis requires JA signalling, while the biosynthesis of the major diterpenoid phytoalexins is regulated by both JA-required and non-JA-required pathways^9^,^10^. Besides, blast-induced accumulation of sakuranetin is known to be compromised in a JA-deficient mutant *cpm2*, while accumulation of diterpenoid phytoalexins remained^9^. Therefore, we focused on the biosynthetic pathway for sakuranetin as a model for JA-required defence responses. In the final step of sakuranetin biosynthesis, the precursor naringenin is converted to sakuranetin by naringenin 7-*O*-methyltransferase (NOMT), which is encoded by *OsNOMT*^11^. We recently reported that *OsNOMT* expression is induced by JA treatment^12^, and jasmonoyl-l-isoleucine (JA-Ile), an active form of jasmonate, is required for the production of sakuranetin^10^. We have previously shown that CuCl_2_ is a potent elicitor to induce sakuranetin production in wild-type plants, while such CuCl_2_-inductive production of sakuranetin is specifically compromised in a JA-Ile deficient mutant *osjar1*^10^. These findings prompted us to speculate that the elucidation of the regulation mechanism underlying *OsNOMT* expression would help in understanding the mechanism underlying JA-required sakuranetin production.

For the production of specialized metabolites, diverse transcriptional factors (TFs) have been characterized as hormone-inducible regulators in various plant species^4^,^13^,^14^. In particular, several TFs have been well-studied and reported to contribute to the production of diterpenoid phytoalexins or expression of their biosynthetic genes in rice^15^,^16^,^17^,^18^. However, regulatory factors responsible for sakuranetin production and/or *OsNOMT* expression remain unclear.

OsMYC2, a basic helix-loop-helix (bHLH) TF in rice, is a homologue of AtMYC2, a well-studied global regulator of JA signalling in Arabidopsis^19^,^20^,^21^. OsMYC2 is involved in JA-required spikelet development by the direct activation of *OsMADS1*^22^. Another study revealed that OsMYC2 interacts with almost all of 14 JASMONATE ZIM-domain (JAZ) proteins in rice via the JAZ-interacting domain, one of which represses JA-inducible *OsMYC2* expression. Furthermore, overexpression of *OsMYC2* causes upregulation of early JA-responsive genes, leading to JA-hypersensitive phenotypes such as bacterial blight resistance^23^. However, the mechanism underlying the activation of the downstream pathway of OsMYC2 remains largely unknown.

Here, we report that JA-induced expression of *OsNOMT* and production of sakuranetin are under the control of OsMYC2. Moreover, we identified two bHLH proteins, OsMYC2-like protein 1 (OsMYL1) and OsMYL2, as interacting partners of OsMYC2. Both OsMYL1 and OsMYL2 enhance the transactivation activity of OsMYC2 by physical interaction, leading to further activation of the *OsNOMT* promoter. Our findings will help in understanding how rice plants modulate the activity of OsMYC2 during JA signalling, leading to the inductive production of sakuranetin as a defence response.

## Results

### Selection of regulatory factor candidates for *OsNOMT* by using transcriptome analysis of JA-deficient plants

To identify JA-dependent transcriptional factors (TFs), which could be possible candidates for the *OsNOMT* regulation in response to JA, we performed transcriptome analysis using CuCl_2_-elicited *cpm2*, a JA-deficient mutant^9^, in comparison with wild-type plants (WT). We used CuCl_2_ as an elicitor to induce sakuranetin production via JA signaling because CuCl_2_ treatment is also known to induce JA production and reveals good reproducibility to elicit these phenomena. Since *OsNOMT* was also shown to be induced within 24 h after CuCl_2_ treatment (see Supplementary Table S1), JA-dependent regulatory factors for *OsNOMT* were expected to precede the upregulation of *OsNOMT*. Therefore, we selected TFs that fulfilled the following criteria: upregulated over 2-fold in either 2 or 6 h after CuCl_2_ treatment in WT and downregulated under 0.5-fold in either 2 or 6 h after CuCl_2_ treatment in *cpm2* when compared with WT. We also used a parameter Q value to obtain a significant number of genes along with an acceptable false discovery rate (FDR) less than 0.05. As a result, various types of 41 TFs comprising bHLH, MYB, NAC, WRKY, and bZIP families were selected (see Supplementary Table S2), and they included the JA-inductive bHLH factor OsMYC2 and MYB factor OsJAmyb. The expression of *OsMYC2* is induced by JA treatment^23^. Several MYB proteins activate flavonoid biosynthetic genes by interacting with bHLH TFs^24^. Thus, we focused on the MYB and bHLH proteins among the selected TFs.

### OsMYC2 functions as the transcriptional activator of the *OsNOMT* promoter

**Figure 1:**
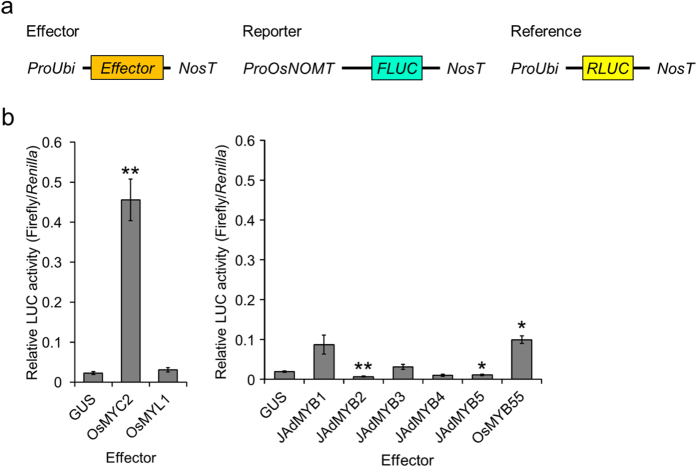
Activation of the *OsNOMT* promoter by JA-induced transcription factors. (**a**) Schematic representation of the vectors used for the transient expression assays. FLUC: firefly luciferase, RLUC: renilla luciferase, ProOsNOMT: 5′ 1000-bp upstream region of the transcription start site of *OsNOMT*, ProUbi: maize ubiquitin promoter, NosT: nopaline synthase terminator. (**b**) Relative luciferase activities in the bombarded rice leaves. Data are presented as FLUC/RLUC ± standard error. n = 3–5. *P < 0.05, **P < 0.01, compared to the data with GUS as the effector.

To investigate the effects of the selected TFs, we analysed their transactivation activity by using the *OsNOMT* promoter-luciferase (LUC) reporter assay. Candidate TFs (OsMYC2, OsMYL1, JA-dependent [JAd] MYB1–MYB5, OsMYB55, and OsJAmyb) were driven by the maize ubiquitin promoter as effectors for their constitutive expression. The luciferase activity assay without the *OsNOMT* promoter revealed that all the effectors, except OsJAmyb, did not activate the reporter activity in a promoter-independent manner (see Supplementary Fig. S1). Then, we performed the luciferase activity assay with one of the selected proteins, except OsJAmyb, as the effector. As a result, 3 proteins, OsMYC2, JAdMYB1, and OsMYB55, were found to activate the promoter activity of *OsNOMT* (Fig. 1). Among these TFs, OsMYC2 exhibited the most robust transactivation activity.

### Impacts of modification of *OsMYC2* expression on *OsNOMT* expression and phytoalexin production

**Figure 2:**
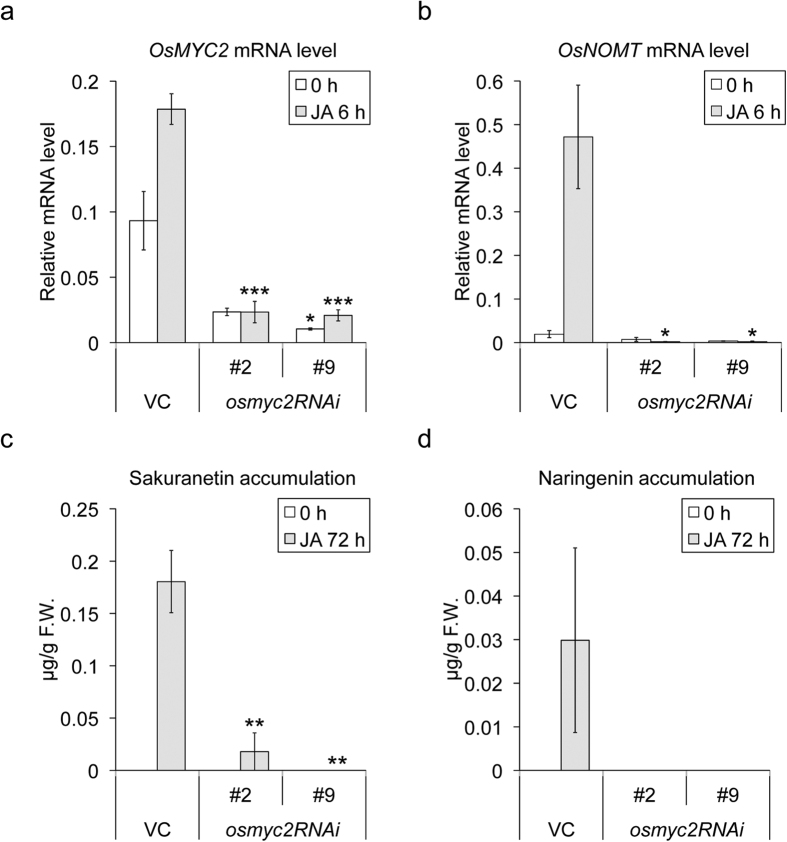
Gene expression and phytoalexin accumulation in the *OsMYC2*-knockdown plants (*osmyc2RNAi*). (**a**,**b**) Expression levels of *OsMYC2* (**a**) and *OsNOMT* (**b**). Total RNA from the rice leaves after 6 h of 500 μM JA treatment (grey bars) or untreated rice leaves (open bars) was extracted. qRT-PCR analysis was performed using the cDNA samples synthesized from the total RNA. (**c**,**d**) Contents of sakuranetin (**c**) and naringenin (**d**) in the rice leaves after 6 h of 500 μM JA treatment (grey bars) or no treatment (open bars). Data are presented as mean ± standard error values. (**a**,**b**) n = 3–4, (**c**,**d**) n = 4–6. *P < 0.05, **P < 0.01, ***P < 0.001, compared to the VC data for each treatment.

MYC2 is known as a central regulator of JA-signalling in some plants^20^,^25^. In rice, OsMYC2 has also been shown to be involved in JA-signalling through interaction with JAZ repressor proteins^23^. The luciferase activity assay clearly demonstrated that OsMYC2 has a positive impact on the expression of *OsNOMT*. To further dissect the effects of OsMYC2 on the expression of *OsNOMT* and production of sakuranetin *in planta*, we generated *OsMYC2*-RNAi knockdown plants (henceforth *osmyc2RNAi*) along with empty vector control (VC) plants. Under basal conditions, the expression level of *OsMYC2* was repressed by 75% and 89% in 2 independent *osmyc2RNAi* lines (#2 and #9), respectively, with no visible phenotypes, compared with the VC plants. Expression of *OsMYC2* was induced by JA treatment in the VC plants, as shown by Uji *et al*.^23^. The induction of *OsMYC2* by JA treatment was not observed in *osmyc2RNAi* lines (Fig. 2a). We next investigated the expression of *OsNOMT* after elicitation. Expression of *OsNOMT* was induced by JA treatment in the VC plants. In the *osmyc2RNAi* lines, JA-inducible expression of *OsNOMT* was totally abolished (Fig. 2b). Quantification of phytoalexin contents revealed that the production of sakuranetin and precursor naringenin induced by JA was repressed in *osmyc2RNAi* lines (Fig. 2c,d). These data indicate that JA-induced induction of *OsNOMT* expression and production of sakuranetin are under the control of OsMYC2 in rice.

### Transcriptional factors that work with OsMYC2 to activate the *OsNOMT* promoter

**Figure 3:**
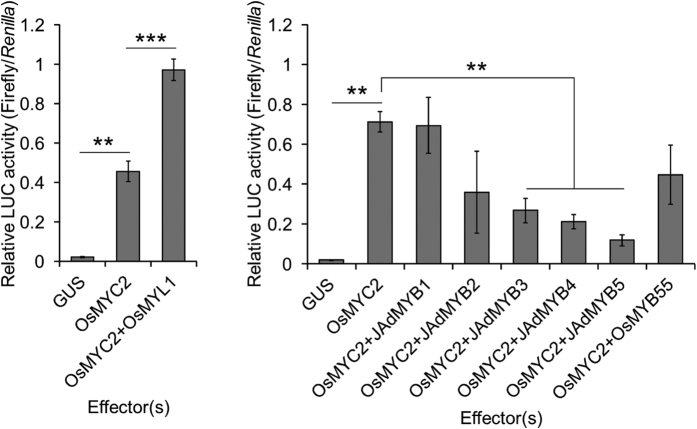
Luciferase activity assay with dual effectors. Relative luciferase activities in the bombarded rice leaves. Data are presented as FLUC/RLUC ± standard error. n = 3–5. **P < 0.01, ***P < 0.001.

The interaction of bHLH and MYB TFs often enhances the transcription of flavonoid biosynthetic genes^24^. To test this point, we next performed transient expression assays with dual effectors. MYB proteins or OsMYL1 was co-introduced with OsMYC2 as effectors. As a result, OsMYL1 further activated the transactivation of *OsNOMT* when co-introduced with OsMYC2, while any MYB TF did not activate transactivation, or rather repressed (Fig. 3). These results indicate that OsMYC2 and OsMYL1, both bHLH proteins, synergistically enhance the transactivation of *OsNOMT*.

**Figure 4:**
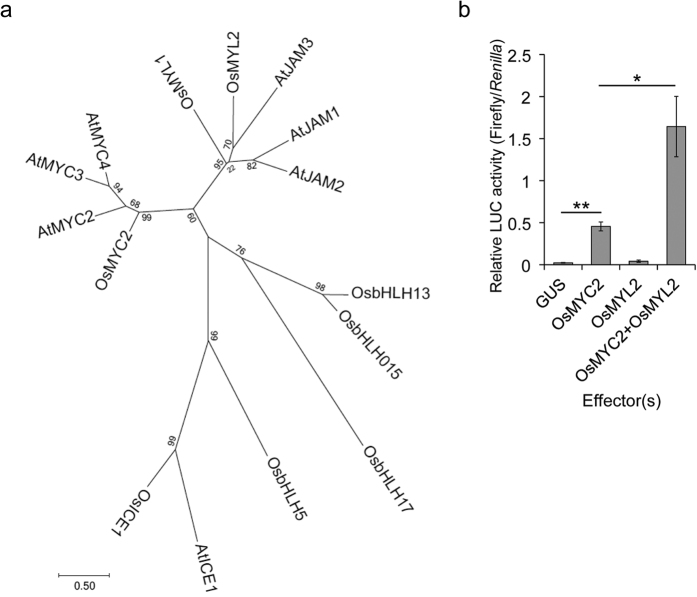
Transactivation activity of OsMYL2. (**a**) Phylogenetic tree of the bHLH transcription factors. We used the JTT+G model to construct the phylogenetic tree. (**b**) Relative luciferase activities in the bombarded rice leaves. Data are presented as FLUC/RLUC ± standard error. n = 5. *P < 0.05, **P < 0.01.

We found another OsMYL protein designated as OsMYL2 that is highly homologous to both OsMYC2 and OsMYL1 (Fig. 4a). Although the expression of *OsMYL2* was not affected by CuCl_2_ in WT in our transcriptome analysis (see Supplementary Table S2), we included this TF in our further analysis because of its similarity to OsMYL1. The luciferase activity assay with OsMYC2 and/or OsMYL2 revealed that OsMYL2 also activated the transactivation of *OsNOMT* when co-introduced with OsMYC2, just as in the case of OsMYL1 (Fig. 4b). We also confirmed that any combination did not affect the reporter activity in an *OsNOMT* promoter-independent manner (see Supplementary Fig. S2).

### OsMYL1 and OsMYL2 as interacting partners of OsMYC2

**Figure 5:**
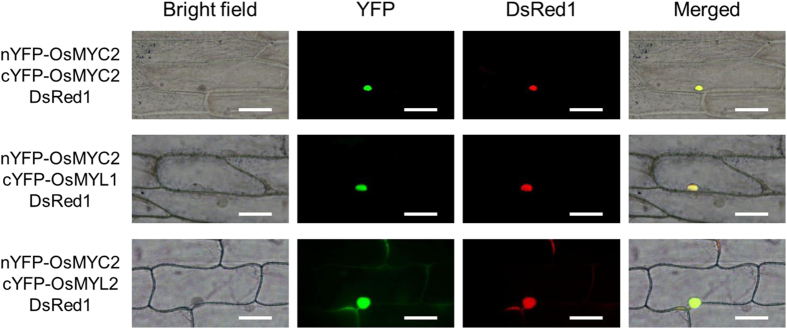
BiFC visualization of OsMYC2-OsMYC2/OsMYL1/OsMYL2 interactions. Onion epidermal cells were co-transfected with constructs encoding the nEYFP-fused proteins, cEYFP-fused proteins, and DsRed1. Images were obtained 24 h after bombardment by using the BX53 microscope system (Olympus). Bars = 100 μm.

Since the helix-loop-helix motif in bHLH proteins has been reported to form dimers^26^, we speculated that OsMYC2 interacts with OsMYL1 and OsMYL2, leading to further activation of the *OsNOMT* promoter. To validate this hypothesis, we performed bimolecular fluorescence complementation (BiFC) assays. Vectors expressing nEYFP-fused protein, cEYFP-fused protein, and DsRed1 (pTH121R^17^) under the control of the CaMV 35S promoter were introduced into onion epidermal cells by particle bombardment. With the co-expression of nYFP-OsMYC2 and cYFP-OsMYC2, c-YFP-OsMYL1, or cYFP-OsMYL2, YFP fluorescence and DsRed1 fluorescence were detected, especially in the nucleus (Fig. 5). However, with the co-expression of nYFP-OsMYC2/cYFP, nYFP/cYFP-OsMYC2, nYFP/cYFP-OsMYL1, or nYFP/cYFP-OsMYL2, only DsRed1 fluorescence was detected (see Supplementary Fig. S3). These data indicate that OsMYC2 interacts with OsMYL1, OsMYL2, and OsMYC2 alone in the nucleus.

**Figure 6:**
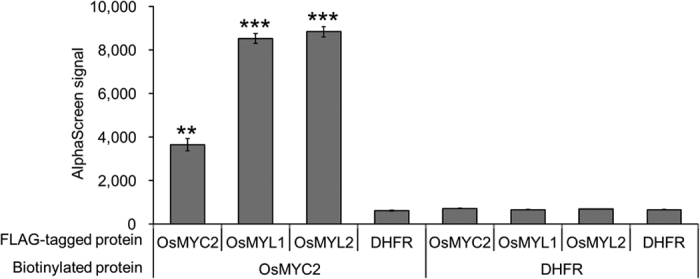
AlphaScreen-based evaluations of the OsMYC2-OsMYC2/OsMYL1/OsMYL2 interactions. Crude translation mixtures of biotinylated His-Bls-OsMYC2 and FLAG-tagged proteins, OsMYC2, OsMYL1, or OsMYL2, were used for the assay. Biotinylated dihydrofolate reductase (DHFR) and FLAG-tagged DHFR were used as the negative controls. Data are presented as mean ± standard error values. n = 3. **P < 0.01, ***P < 0.001, compared to the data for biotinylated DHFR and FLAG-tagged DHFR.

Next, we performed *in vitro* protein-protein interaction assays by using the amplified luminescent proximity homogeneous assay (AlphaScreen). Since we failed to express tagged OsMYC2 translated from original coding sequence (CDS), we used optimized CDS by synonymous substitution using codon usage of *Arabidopsis thaliana* (see *Optimized synthetic CDS of* OsMYC2 online). His-Bls-OsMYC2 (Bls: biotin ligase site^27^) and FLAG-tagged OsMYC2, OsMYL1, and OsMYL2 were expressed using the wheat germ expression system (see Supplementary Fig. S4). The AlphaScreen assay using biotinylated His-Bls-tagged and FLAG-tagged proteins revealed that OsMYC2 physically interacts with OsMYL1, OsMYL2, and OsMYC2 alone *in vitro* (Fig. 6).

### Transactivation activity of OsMYC2 enhanced by interaction with OsMYL1 or OsMYL2

**Figure 7:**
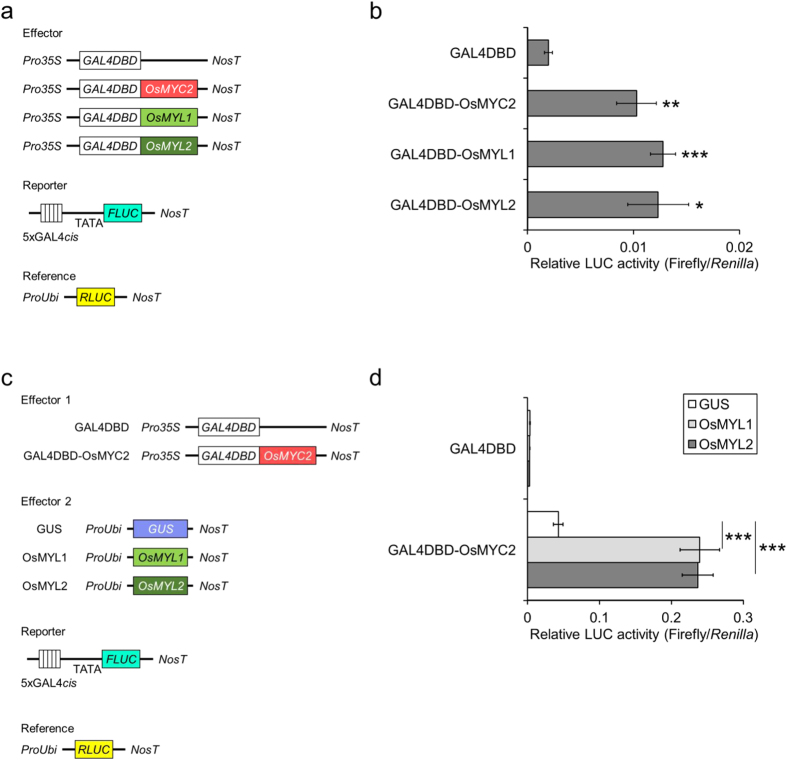
Transcriptional activities of OsMYC2, OsMYL1, and OsMYL2. (**a**) Schematic representation of the vectors used for the transient expression assays. GAL4DBD: GAL4 DNA-binding domain, FLUC: firefly luciferase, RLUC: renilla luciferase, Pro35S, CaMV 35 S promoter, ProUbi: maize ubiquitin promoter, NosT: nopaline synthase terminator. (**b**) Relative luciferase activities in the bombarded rice leaves. Data are presented as FLUC/RLUC ± standard error. n = 6. *P < 0.05, **P < 0.01, ***P < 0.001, compared to the data for GAL4DBD. (**c**) Schematic representation of the vectors used for the transient expression assays with GAL4DBD-OsMYC2 co-transfected with GAL4DBD-free OsMYL1/OsMYL2. (**d**) Relative luciferase activities in the bombarded rice leaves. Data are presented as FLUC/RLUC ± standard error. n = 6. ***P < 0.001.

Protein-protein interaction sometimes strikingly affects the function of proteins. This prompted us to test whether the interaction of OsMYC2 with OsMYL1 and OsMYL2 is the casual event of the enhanced transactivation. We investigated how OsMYL1 and OsMYL2 act on the enhancement by using the artificial GAL4 DNA-binding domain (DBD)-fused system^28^, that is, TFs were forced to interact with the promoter region (Fig. 7a). When OsMYL1 or OsMYL2 was fused to GAL4DBD, they exhibited transactivation activity similar to the activity detected in OsMYC2^22^ (Fig. 7b). Furthermore, co-expression of OsMYL1 or OsMYL2 with GAL4DBD-OsMYC2 resulted in obvious enhancement of the transactivation ability of OsMYC2 (Fig. 7c,d). These data indicate that OsMYL1 and OsMYL2 synergistically activate the transactivation activity of OsMYC2 by physical interaction.

## Discussion

Jasmonic acid (JA) and its derivatives contribute greatly to defence responses in the plant kingdom, including production of defence-related specialized metabolites that are strictly regulated by various transcriptional factors (TFs) through the JA signalling pathway. Here, we demonstrated that the bHLH TF OsMYC2 plays a pivotal role in the JA-induced expression of *OsNOMT* that is enhanced by physical interaction with OsMYL1 and OsMYL2, presumably leading to the consequent accumulation of a flavonoid phytoalexin, sakuranetin, during rice defence responses. Repression of *OsMYC2* expression strikingly impaired JA-induced *OsNOMT* expression and production of sakuranetin and naringenin (Fig. 2). We also found that CuCl_2_-induced sakuranetin production was impaired in *osmyc2RNAi* lines, although CuCl_2_-induced expression of *OsNOMT* itself was comparable to that in the VC plants (see Supplementary Fig. S5). These results imply that the sakuranetin biosynthetic pathway, specifically the upper flavonoid biosynthetic pathway, would be highly dependent on OsMYC2, while the knockdown of *OsMYC2* did not affect the inductive expression of *OsNOMT* after CuCl_2_ treatment. Hence, the expression of *OsNOMT* would also be induced in an OsMYC2-independent manner.

OsMYL1 and OsMYL2 have higher homology to jasmonate-associated MYC2-like proteins (AtJAMs) 1 to 3, rather than OsMYC2 or AtMYC2 (Fig. 4a). OsMYC2 and AtMYC2 are categorized as IIIe group bHLH TFs, while OsMYL1, OsMYL2, and AtJAMs are categorized as IIId group bHLH TFs^29^. AtJAMs are negative regulators of JA signalling^30^. Another study revealed that AtJAM1 is a transcriptional repressor that competes with AtMYC2 by sharing the same binding elements. In addition, AtJAM1 does not interact with AtMYC2^31^. Meanwhile, we found that OsMYL1 and OsMYL2 are the collaborators of OsMYC2 that enhance the transactivation activity of OsMYC2 by physical interaction (Figs 3, 4, 5, 6 and 7). Probably, OsMYL1 and OsMYL2 enhance the transactivation activity of OsMYC2 by forming heteromers (Figs 5, 6 and 7), which may lead to the further activation of JA signalling. However, the actual forms of the homomer and heteromers that may be critical for their functions remain unclear. Our effector assay revealed that OsMYL1 and OsMYL2 themselves were not able to activate the *OsNOMT* promoter without being forced to bind to DNA as the GAL4DBD-fused proteins, indicating that formation of the OsMYC2 and OsMYL heteromer is important for their transactivation ability, possibly by activating OsMYC2 by altering protein structure.

The relationships between AtMYC2 and AtJAMs are totally different from those between OsMYC2 and OsMYL1 or OsMYL2, which may cause the differences in the regulatory mechanisms of the JA-induced defence responses between monocots and dicots. Identification of the important region(s) of OsMYC2, OsMYL1, and OsMYL2 protein for transcriptional activation activities and protein-binding abilities would be required to elucidate these differences in the future. Besides, we suggest apparent difference between OsMYL1 and OsMYL2 on the basis of our transcriptome analysis; expression of *OsMYL1* as well as *OsMYC2* was induced in a JA-dependent manner, while expression of *OsMYL2* was constant (see Supplementary Table S2). Whether optimal use of the OsMYC2-OsMYL1 and OsMYC2-OsMYL2 heteromers *in planta* is required for appropriate induction of *OsNOMT* is an interesting question.

DNA-binding ability is one of the most essential functions of the TFs, as well as transactivation activity. To evaluate the DNA-binding ability of OsMYC2, or the heteromers formed with OsMYL1 or OsMYL2, we also tried to find the promoter region of *OsNOMT* responsible for OsMYC2-dependent activation in this study. However, even though most of the promoter region, except for the TATA box located at −34 to −27 bp from the transcriptional start site, was deleted, there was still OsMYC2-dependent activation in the deletion series; consequently, we could not define the *cis*-element bound by OsMYC2 (see Supplementary Fig. S6 and Supplementary Methods). Since the DNA-binding ability of OsMYC2 would be affected by physical interaction with OsMYL1 and OsMYL2, co-introduction of OsMYC2 and OsMYLs should be tested further. Uji *et al*. reported that OsMYC2 binds to a specific E-box sequence of the *OsJAZ10* promoter *in vivo* by using the chromatin immunoprecipitation strategy^23^. Therefore, *in vivo* experimental approaches would be required to understand OsMYC2 binding on the *OsNOMT* promoter. Alternatively, it might be possible that OsMYC2-mediated induction of *OsNOMT* expression is not regulated by TF(s) directly binding to the promoter region but is regulated in the post-transcriptional stage.

Sakuranetin, a biologically important phytoalexin in rice, is a good subject for elucidating JA signalling because of its high JA-required accumulation, whereas production of diterpenoid phytoalexins occurs via both JA-required and non-JA-required pathways^9^,^10^. Deciphering JA signalling mediated by OsMYC2, OsMYL1, and OsMYL2 would help in understanding the regulation of the flavonoid biosynthetic pathway for naringenin and sakuranetin. It could be possible to generate plants that overproduce sakuranetin by heterologous expression of *OsNOMT*, and further attempts to use OsMYC2 and its collaborators, OsMYL1 and OsMYL2, for enhancing *OsNOMT* expression and the early flavonoid biosynthetic pathway could be a potential method of generating sakuranetin-biofortified plants.

## Methods

### Plant materials

For the luciferase activity assays, we used the *japonica* type rice *Oryza sativa* L. ‘Nipponbare’. For the transcriptome analysis, we used a JA-deficient mutant, *cpm2*^9^, and its genetic background, *Oryza sativa* L. ‘Nihonmasari’ as WT. For the gene expression analyses and phytoalexin quantification analyses, we used RNAi-based *OsMYC2*-knockdown plants (*osmyc2RNAi*) under the control of the *Zea mays* polyubiquitin promoter and VC plants transformed with the empty vector (pANDA^32^), with their genetic background, *Oryza sativa* L. ‘Nihonmasari’, as WT.

To grow the rice plants, the seeds were sterilized with 70% ethanol for 2 min, followed by treatment with 1% sodium hypochlorite solution (Kanto Chemical) for 20 min; the seeds were then washed with autoclave-sterilized water. The surface-sterilized seeds were incubated on 0.5% agar medium in a chamber (14-h light and 10-h darkness at 28 °C). Rice transformation was performed according to the method reported by Toki *et al*.^33^.

### Cloning

Standard methods for cloning were used. PCR-amplified DNA fragments were sequenced after cloning into the vectors. The vectors used in this study and primers used for the cloning are listed in Supplementary Tables S3 and S4, respectively.

#### pUbi-RLUC

The CDS of renilla luciferase from pRL (Promega, WI, USA) was PCR-amplified and cloned into the pENTR/D-TOPO vector (Invitrogen, CA, USA). The CDS was then inserted into the pUbi-RfA-Tnos vector^15^ by using the LR reaction (Gateway^®^).

#### pGL4-OsNOMT_1000_

The 5′ 1-kb upstream region of the *OsNOMT* promoter, extending from the transcriptional start site, was PCR-amplified and cloned into the pZErO2 vector (Invitrogen, CA, USA). The promoter region was extracted and inserted into multi-cloning sites of pGL4.10-Tnos, which the SV40 late poly (A) signal of pGL4.10 (Promega, WI, USA) was converted to the nopaline synthase terminator (Tnos) at the *Bam*HI and *Xba*I sites, to obtain pGL4-OsNOMT_1000_.

#### pUbi-JA-dependent MYB1, pUbi-JA-dependent MYB2, pUbi-JA-dependent MYB3, pUbi-JA-dependent MYB4, pUbi-JA-dependent MYB5, pUbi-OsJAmyb, pUbi-OsMYB55, pUbi-OsMYC2, pUbi-OsMYL1, and pUbi-OsMYL2

Each CDS was PCR-amplified and cloned into the pENTR/D-TOPO vector. The CDS was then inserted into the pUbi-RfA-Tnos vector by using the LR reaction. The CDS of OsMYL1 was 39 bp longer than the RAP-DB annotation (see *CDS of* OsMYL1 *described in this article* online).

#### pANDA-OsMYC2

The 748-bp trigger region of *OsMYC2*, which consists of 336 bp from the 3′ UTR and 412 bp from the 3′ CDS, was PCR-amplified and cloned into the pENTR/D-TOPO vector. The trigger region was then inserted into the pANDA vector^32^ by using the LR reaction.

#### pnYOsMYC2, pcYOsMYC2, pcYOsMYL1, and pcYOsMYL2

Each CDS, cloned into pENTR/D-TOPO as described above, was inserted into the pnYGW or pcYGW vector^34^ by using the LR reaction.

#### pEU-E01-6His-Bls-GW-STOP

We performed the inverse overlap extension PCR by using pEU-E01-GW as the template^35^. Amplified DNA fragments were self-ligated using the In-Fusion system (TaKaRa Bio USA, CA, USA) to obtain pEU-E01-His-Bls-GW-STOP.

#### pEU-E01-6His-Bls-optOsMYC2 and pEU-E01-FLAG-optOsMYC2

The optimized CDS of *OsMYC2* was synthesized using the GeneArt^®^ Strings™ DNA Fragments service (Invitrogen, CA, USA), PCR-amplified in twice, and cloned into the pENTR/D-TOPO vector. The CDS was then inserted into the pEU-E01-6His-Bls-GW-STOP or pEU-E01-FLAG-GW-STOP^35^ vector by using the LR reaction.

#### pEU-E01-FLAG-OsMYL1 and pEU-E01-FLAG-OsMYL2

Each CDS, cloned into pENTR/D-TOPO as described above, was inserted into the pEU-E01-FLAG-GW-STOP vector by using the LR reaction.

#### p35S-GAL-OsMYC2, p35S-GAL-OsMYL1, and p35S-GAL-OsMYL2

Each CDS was PCR-amplified and inserted into 430T1.2^36^ by using the In-Fusion system (TaKaRa Bio USA, CA, USA).

#### Luciferase activity assay

Six- or 7-day-old rice leaves were excised from the sheath, acclimated overnight, and then used for particle bombardment.

Effector plasmids were used to constitutively express the TFs as effectors under the control of the *Z. mays* polyubiquitin promoter in the rice leaves. pUbi-GUS^15^ was used as the control. Constructs, in which the promoter region was fused to the firefly luciferase (*FLUC*) gene, were used as reporter plasmids. pUbi-RLUC, in which renilla luciferase (*RLUC*) was under the control of the *Z. mays* polyubiquitin promoter, was used as the internal control. The PDS-1000 He Biolistic Particle Delivery System (Bio-Rad, CA, USA) was used for the particle bombardment.

For particle bombardment, gold particles (0.6 mg and 1.6 μm in diameter; Bio-Rad, CA, USA) were coated with 1 μg of the reporter plasmid, 0.5 μg of pUbi-RLUC, and effector plasmid(s) (0.5 μg each). The plasmid-coated particles were bombarded at 1100 psi and 9-cm distance. After the bombardment, the rice leaves were incubated for 24 h in a chamber (24-h light at 28 °C) before the luciferase assays. The assays were performed as described previously^17^. The ratio of luciferase activity (Firefly LUC/*Renilla* LUC) was calculated to normalize the values in each assay.

### Transcriptome analysis

Total RNA was extracted from 1 mM of the CuCl_2_-treated plants by using the RNeasy plant mini kit (QIAGEN). Microarray analyses were performed as described previously^15^. Three biological replicate sample sets were analysed. Data normalization and statistical analyses were performed using the Subio Platform with Basic Plug-in (Subio Inc., Kagoshima, Japan). Transcriptome data have been deposited in the Gene Expression Omnibus in NCBI (ID: GSE87698).

### Expression analyses

Prior to the experiments, the transgenic plants were selected by PCR amplification of the hygromycin phosphotransferase (*HPT*) transgene with the primers 5′-ATGAAAAAGCCTGAACTCACCGCGACGTCTGTC-3′ and 5′-CTATTCCTTTGCCCTCGGACGAGTGCTG-3′. Ten-day-old rice leaves were excised from the sheath, acclimated overnight (light at 28 °C), and then used for the subsequent experiments. The rice leaves were elicited with 500 μM of JA or CuCl_2_ for 6 h. Leaves collected before elicitation were used as the control. Total RNA extracted using the Maxwell^®^ 16 LEV Plant RNA Kit (Promega, WI, USA) was reverse-transcribed with the PrimeScript™ RT reagent Kit with gDNA Eraser (Perfect Real Time) (TaKaRa BioInc., Japan). The synthesized cDNA was used to perform qRT-PCR. qRT-PCR was performed using the Power SYBR^®^ Green PCR Master Mix (Applied Biosystems, CA, USA) and Applied Biosystems 7300 Real-Time PCR System (Applied Biosystems). To calculate the relative mRNA levels, raw data from qRT-PCR analysed using the standard curve method were normalized by the raw data of the ubiquitin domain-containing protein gene (*OsUBQ*). Sequences of the primers used for the expression analyses are provided in Supplementary Table S5.

### Quantification of phytoalexins and naringenin accumulated in the leaves

Prior to the experiments, the transgenic plants were selected by PCR amplification of the *HPT* transgene with the primers described above. Ten-day-old rice leaves were excised from the sheath, acclimated overnight (light at 28 °C), and then used for the subsequent experiments. The rice leaves were elicited with 500 μM of JA or CuCl_2_ for 72 h, soaked in phytoalexin extraction solvent (ethanol:water:acetonitrile:acetic acid, 79:13.99:7:0.01, v/v), and incubated for 24 h at 4 °C to extract the phytoalexins and naringenin. Leaves collected before elicitation were used as the control. The samples were then centrifuged (4 °C, 15 min, and 16,000 *g*). The supernatant was collected and subjected to phytoalexin and naringenin measurement by using liquid chromatography-electrospray ionization-tandem mass spectrometry (LC-ESI-MS/MS) as described previously^37^.

### BiFC assay

Constructs encoding the nEYFP-fused proteins, cEYFP-fused proteins, and DsRed1 were introduced into onion epidermal cells by particle bombardment with the PDS-1000 He Biolistic Particle Delivery System (Bio-Rad, CA, USA). Gold particles (0.6 mg and 1.6 μm in diameter; Bio-Rad, CA, USA) were coated with 0.5 μg of each vector for each bombardment. The plasmid-coated particles were bombarded at 1,100 psi and 9-cm distance. The bombarded cells were incubated at 25 °C in darkness for 24 h. YFP and DsRed1 fluorescence was visualized using the BX53 microscope system (Olympus, Japan). The U-FBNA filter cube (excitation filter, BP470–495 nm; dichromatic mirror, DM505 nm; and suppression filter, BA510–550 nm) and U-FGW cube (excitation filter, BP530–550 nm; dichromatic mirror, DM570 nm; and suppression filter, BA575IF nm) were used for detecting YFP and DsRed1 fluorescence, respectively.

### Expression of recombinant proteins

We used the wheat germ cell-free system to express the recombinant proteins^38^,^39^. For expression, the CDS of *OsMYC2* was optimized by synonymous substitution using codon usage of *Arabidopsis thaliana* and synthesized. The expressed His-Bls-OsMYC2 protein was biotinylated before testing the interaction with FLAG-tagged protein for detection.

### AlphaScreen-based protein-protein interaction assays

The AlphaScreen assays with the recombinant proteins described above were performed as described previously^40^, with several modifications. The AlphaScreen assays were performed using a total volume of 15 μL containing 100 mM Tris-HCl (pH8.0), 100 mM NaCl, 0.1% Tween20, 1 mg/mL BSA, 1 μL biotinylated proteins, and FLAG-tagged proteins at 25 °C for 1 h in a 384-well Optiplate (PerkinElmer). Using the AlphaScreen IgG (ProteinA) detection kit (PerkinElmer) instruction manual, 10 μL of the detection mixture containing 100 mM Tris-HCl (pH 8.0), 100 mM NaCl, 0.1% Tween20, 1 mg/mL BSA, 5 μg/mL anti-DYKDDDDK antibody (1E6, Wako Pure Chemical Industries, Ltd. Osaka, Japan), 0.1 μL streptavidin-coated donor beads, and 0.1 μL Protein A-coated acceptor beads were added to each well of the 384-well Optiplate, followed by incubation at 25 °C for 1 h. Luminescence was analysed using the AlphaScreen detection program.

### Statistical analysis

Data are presented as mean ± standard error values. For all the experiments, except transcriptome analysis, statistical analysis was performed using the two-tailed, unpaired Welch’s *t*-test. Significance was defined as P < 0.05 (*), P < 0.01 (**), or P < 0.001 (***). For the transcriptome analysis, statistical testing was performed as described in *Transcriptome analysis*. Significance was defined as false discovery rate (FDR) <0.05.

## Additional Information

**Accession codes:** RAP-DB (http://rapdb.dna.affrc.go.jp/) gene IDs for the sequences described in this study are as follows: JAdMYB1 (Os01g0702700), JAdMYB2 (Os12g0564100), JAdMYB3 (Os01g0192300), JAdMYB4 (Os05g0429900), JAdMYB5 (Os08g0549000), OsJAmyb (Os11g0684000), OsMYB55 (Os05g0553400), OsMYC2 (Os10g0575000), OsMYL1 (Os01g0705700), OsMYL2 (Os01g0235700), OsNOMT (Os12g0240900), and OsUBQ (Os10g0542200).

**How to cite this article:** Ogawa, S. *et al*. OsMYC2, an essential factor for JA-inductive sakuranetin production in rice, interacts with MYC2-like proteins that enhance its transactivation ability. *Sci. Rep.* **7**, 40175; doi: 10.1038/srep40175 (2017).

**Publisher's note:** Springer Nature remains neutral with regard to jurisdictional claims in published maps and institutional affiliations.

## Supplementary Material

Supplementary Information
