# Supplementary material for: OsMYC2, an essential factor for JA-inductive sakuranetin production in rice, interacts with MYC2-like proteins that enhance its transactivation ability
Source: Sci Rep. 2017 Jan 9;7:40175. doi: 10.1038/srep40175 (PMC5220304; doi:10.1038/srep40175)
Supplement: Supplementary Information [file srep40175-s1.pdf]

Supplementary information

OsMYC2, an essential factor for JA-inductive sakuranetin production in rice, interacts with MYC2-like proteins that enhance its transactivation ability

Satoshi Ogawa[1], Koji Miyamoto[2], Keiichirou Nemoto[3], Tatsuya Sawasaki[3], Hisakazu Yamane[2], Hideaki Nojiri[1], and Kazunori Okada[1, *]

Affiliation

[1]Biotechnology Research Center, The University of Tokyo, Bunkyo-ku, Tokyo, Japan

[2]Department of Biosciences, Teikyo University, Utsunomiya, Tochigi, Japan

[3]Proteo-Science Center, Ehime University, Matsuyama, Ehime, Japan

*Corresponding author: E-mail, ukazokad@mail.ecc.u-tokyo.ac.jp; Fax: +81-35841-8030

Supplementary methods

Plant materials

See *Plant materials* in the main text.

Cloning

Standard methods for cloning were used for the construction. PCR-amplified DNA fragments were sequenced after cloning into vectors. The vectors referred to in the *supplementary methods* and primers used for the cloning are listed in Supplementary Tables S6 and S7, respectively.

pGL4-OsNOMT$_{250}$, *pGL4-OsNOMT*$_{150}$, *and pGL4-OsNOMT*$_{50}$: The 5′ 250-, 150-, or 50-bp upstream region of the *OsNOMT* promoter, extending from the transcriptional start site, was PCR-amplified and cloned into the pZErO2 vector (Invitrogen, CA, USA). Each promoter region was extracted and inserted into the multi-cloning sites of pGL4.10-Tnos (see the main text).

pGL4-OsNOMT$_{250\text{-}26}$: The region -250 to -26 bp from the transcriptional start site of *OsNOMT* was PCR-amplified and inserted into *Eco*RV-digested pGL4.10-Tnos.

Luciferase activity assay

See *Luciferase activity assay* in the main text.

Optimized synthetic CDS of *OsMYC2*.

ATGAACCTCTGGACCGATGATAACGCTTCTATGATGGAAGCTTTTATGGCTTCTGCTGAT
CTCCCTGCTTTTCCTTGGGGTGCTGCTTCTACCCCTCCTCCACCACCTCCACCTCCACAT
CATCATCACCAACAGCAGCAACAGCAAGTTCTTCCTCCTCCTGCTGCTGCACCAGCTGCT
GCTGCTTTTAATCAGGATACTCTCCAGCAGAGGCTCCAGTCTATTATCGAGGGATCTAGA
GAGACTTGGACCTACGCTATCTTCTGGCAGTCATCTATCGATGTGTCTACCGGTGCTTCT
CTTCTCGGATGGGGTGATGGATATTACAAGGGATGTGATGATGATAAGAGGAAGCAGAGG
TCATCTACCCCTGCTGCAGCTGCTGAACAAGAGCATAGAAAGAGAGTGCTCAGGGAACTC
AACTCTCTTATCGCTGGTGCTGGTGCTGCTCCTGATGAAGCTGTTGAAGAAGAGGTGACC
GATACCGAGTGGTTCTTCCTTGTGTCTATGACCCAGTCTTTCCCTAACGGACTTGGACTT
CCTGGACAGGCTCTTTTTGCTGCTCAACCTACTTGGATCGCTACCGGACTTTCTTCTGCT
CCTTGCGATAGAGCTAGACAGGCTTACACTTTCGGACTCAGGACTATGGTTTGCCTTCCT
CTCGCTACTGGTGTTCTCGAGCTTGGATCTACCGATGTGATCTTCCAGACCGGTGATTCT
ATCCCTAGAATCAGGGCTCTCTTCAACCTCAGTGCTGCAGCAGCTTCTTCTTGGCCTCCT
CATCCAGATGCTGCTTCAGCTGATCCTTCTGTTCTTTGGCTTGCTGATGCTCCTCCTATG
GATATGAAGGATTCTATCTCTGCTGCTGATATCTCTGTGTCTAAGCCTCCTCCTCCTCCA
CCTCATCAAATCCAGCATTTCGAGAACGGATCTACCTCTACCCTTACCGAGAACCCTTCT
CCTTCTGTGCATGCTCCTACACCTTCTCAACCTGCAGCTCCTCCACAAAGACAACAACAA
CAGCAACAATCTTCTCAGGCTCAGCAGGGACCTTTCAGAAGAGAGCTTAACTTCTCTGAT
TTCGCTTCTAACGGTGGTGCTGCTGCTCCACCATTTTTCAAGCCTGAGACTGGTGAGATC
CTCAACTTCGGAAACGATTCTTCATCTGGAAGAAGAAACCCTTCACCAGCTCCACCAGCA
GCTACAGCTTCTCTTACTACTGCTCCTGGATCACTCTTCTCACAGCACACTCCTACTCTT
ACCGCTGCTGCTAACGATGCTAAGTCTAACAACCAGAAAAGATCTATGGAAGCTACCTCT
AGGGCTTCTAACACCAACAACCATCCTGCTGCAACCGCTAACGAGGGAATGCTCTCTTTT
TCATCTGCTCCAACCACCAGGCCTTCTACTGGAACTGGTGCTCCTGCTAAGTCAGAGTCT
GATCACTCTGATCTCGAGGCTTCAGTGAGAGAGGTTGAGTCATCTAGAGTTGTGGCACCT
CCTCCAGAGGCTGAGAAAAGACCTAGAAAAAGAGGTAGGAAGCCTGCTAACGGAAGGGAA
GAACCTCTCAACCATGTGGAAGCTGAGAGGCAGAGAAGAGAGAAGCTCAACCAGAGATTC
TACGCTCTCAGAGCTGTGGTGCCTAACGTGTCAAAGATGGATAAGGCTTCACTCCTCGGA
GATGCTATCTCTTACATCAACGAGCTTAGGGGAAAGCTCACCGCTCTCGAGACTGATAAG
GAAACCTTGCAGTCTCAGATGGAATCTCTCAAGAAAGAAAGGGATGCTAGGCCTCCTGCT
CCTTCAGGTGGTGGTGGTGATGGTGGTGCTAGATGTCATGCTGTTGAGATCGAGGCTAAG
ATCCTTGGACTCGAGGCTATGATTAGAGTGCAGTGCCACAAGAGAAACCACCCAGCTGCT
AGACTTATGACCGCTCTTAGAGAACTCGATCTCGATGTGTACCACGCTTCTGTGTCTGTG
GTGAAGGATCTCATGATCCAGCAAGTGGCTGTGAAGATGGCTTCTAGGGTGTACTCTCAG
GATCAGTTGAACGCTGCTCTCTACACCAGAATCGCTGAACCTGGTACTGCTGCTAGATGA

CDS of *OsMYL1* described in this article.

ATGTCGTGGTCCGAGACGGACGCCGCGCTGTTCGCGGCGGTGCTGGGGCACGACGCGGCG
CATCACCTGGCCACCACGCCGCCGCACCTCGACGCGCCGGAGGGGTCGCCGTCGTCGGCC
GAGCTGCAGGCGAGCCTGCACGACCTCGTCGAGCGGCAGGGAGGGGCCTGGACGTACGGC
ATCTTCTGGCAGGAGTCCCGCGGCGCCGGTGCCGCCAGCGGCCGCGCTGCCCGCGCCGTG
CTCGGCTGGGGCGACGGCCACTGCCGCGACGGTGCCGGACACGGGGAGGTCGGCGCCGCG
GAGAGGAGCGTGGCGCGGAAGCGCGTGCTGCTGCGGCTGCACGCGCTGTACGGAGGCGGG
GATGAGGATGGCGCCGACTACGCGCTCCGGCTGGACCGGGTCACCGGCGCCGAGATGTAC
TTCCTGGCGTCCATGTACTTCTCCTTCCCGGAGGGCTCGGGCGGACCGGGCCGCGCTCTG
GCCTCCGGCCGCCACGCCTGGGCGGACGTAGACCCCCACCCTTCCGGCTCCGGTAGCGCG
CCAGGGTGGTACGTTCGCTCGTCTCTCGCCCAGTCCGCGGGGTTACGCACCGTCGTCTTC
CTCCCGTGCAAGGGCGGCGTTCTCGAGCTCGGTTCCGTCGTAGCCATCCGCGAGACCCCC
GAGGTCTTGCGCGCCATCCAATCTGCCATGCGCGCCGTGCCAGCTCCGCCGGAAGATTTC
ATGAGAATCTTCGGCAAGGATCTCTCACCCGGCCGACCGTCCCAGCCCATGGGATGCGAC
GCCCCATGGACGCCGCGGCTCGTTGTCCAAACCACGCCGGTGCGCCCAGCCAAGAAGGAG
GTGGTCAAGGCGAAACCAGCCGAGCCCCCCAAGAGCTTGGACTTCTCCAAGGCGAACGTG
CAGGAACAGGCCGGCGGCCAGGAGCGGCGGCCGCGGAAGCGTGGGCGCAAGCCGGCGAAC
GGGCGGGAGGAGCCGCTGAACCACGTGGAGGCGGAGCGGCAGCGGCGGGAGAAGCTGAAC
CAGAGGTTCTACGCGCTGCGCGCGGTGGTGCCCAAGATCTCCAAGATGGACAAGGCGTCC
CTGCTCAGCGACGCCATCGCGTACATCCAGGAGCTGGAGGCCCGGCTCAGGGGCGACGCG
CCCGTGCCCGCGCGGGCGGATGGGCCGGCCGTGGAGGTGAAGGCAATGCAGGACGAGGTG
GTGCTGCGCGTGACCACGCCCCTGGACGAGCACCCCATCTCCAGGGTGTTCCACGCCATG
AGGGAATCCCAGATCAGCGTCGTGGCGTCGGACGTGGCGGTGTCGGACGACGCCGTCACG
CACACGCTCATGGTGCGGTCGGCCGGGCCGGAGCGGCTCACGGCGGAGACGGTGCTCGCG
GCGATGTCGCGGGGGGTGAGCGTCACCACTCCCTCCCCGTGA

Underlined region is annotated as intron in RAP-DB, but not in MSU libraries.

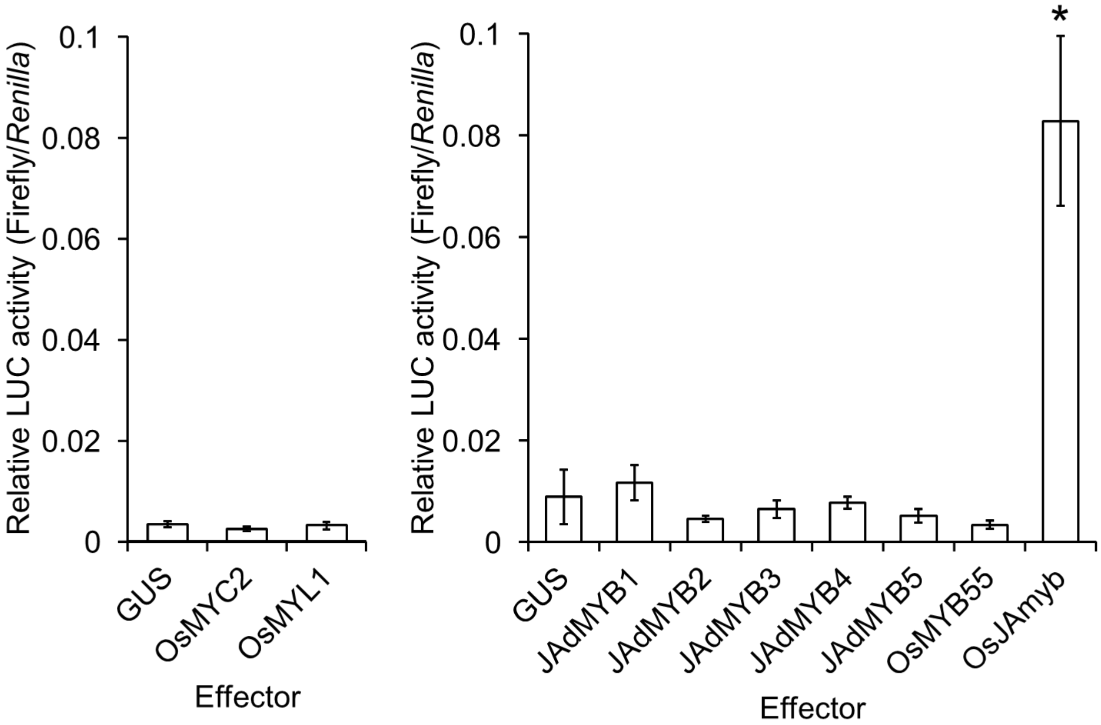

Supplementary Figure S1 ***OsNOMT* promoter-independent effects of JA-induced transcription factors.** Relative luciferase activities in the bombarded rice leaves. Data are presented as FLUC/RLUC ± standard error. n = 3–5. *P < 0.05, compared to the data with GUS as the effector.

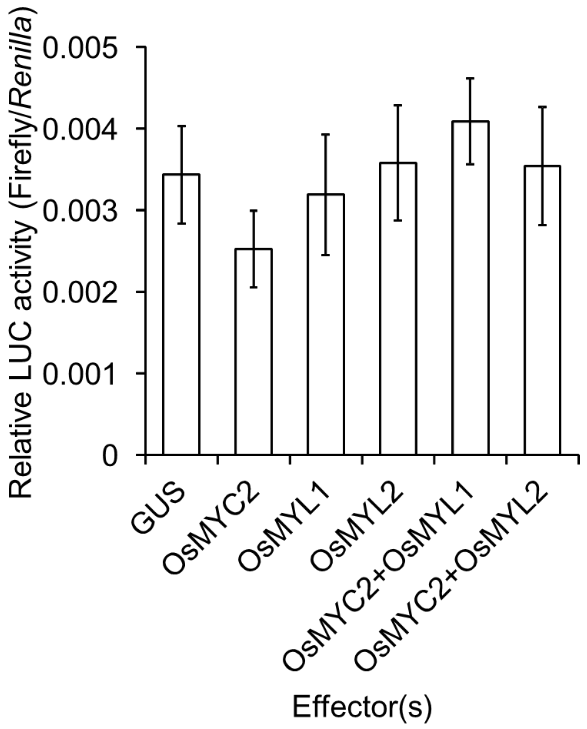

Supplementary Figure S2 ***OsNOMT* promoter-independent effects of OsMYC2, OsMYL1, and OsMYL2.** Relative luciferase activities in the bombarded rice leaves. Data are presented as FLUC/RLUC ± standard error. n = 4.

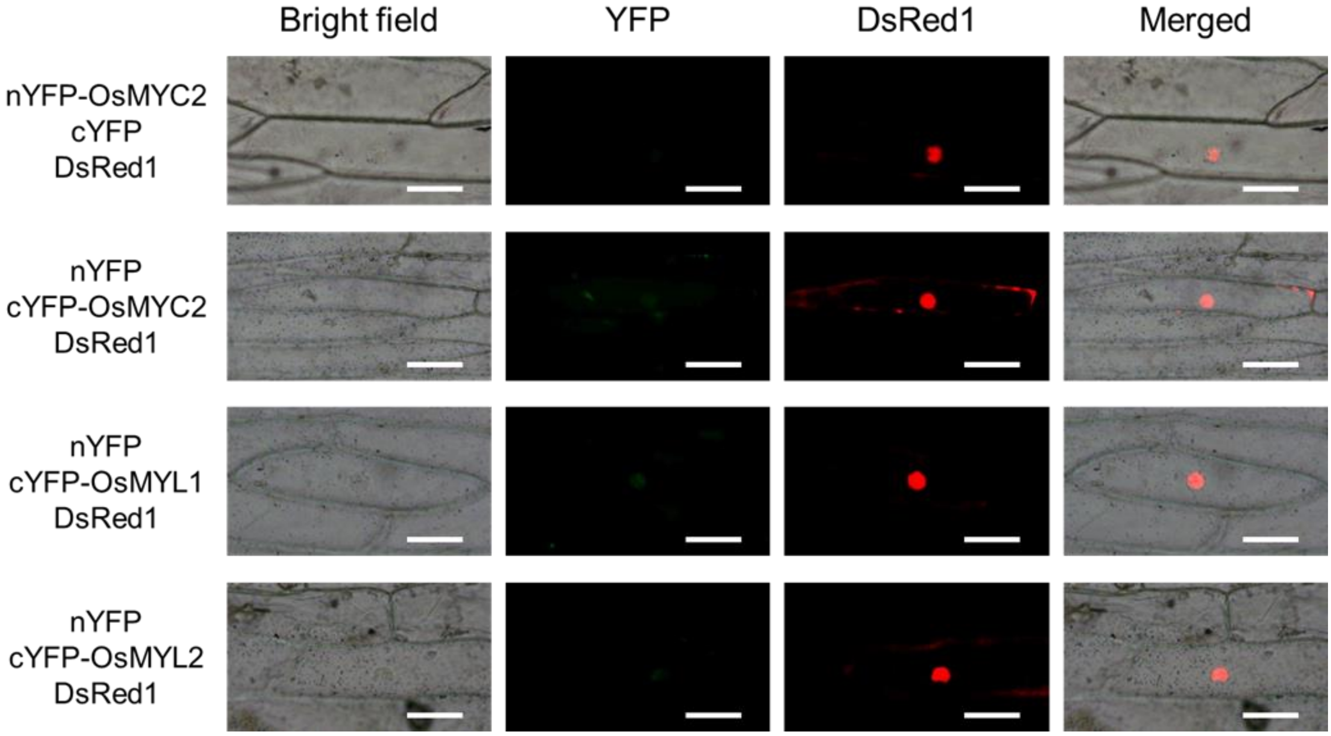

Supplementary Figure S3 **Negative controls of BiFC assays.** Bars = 100 µm.

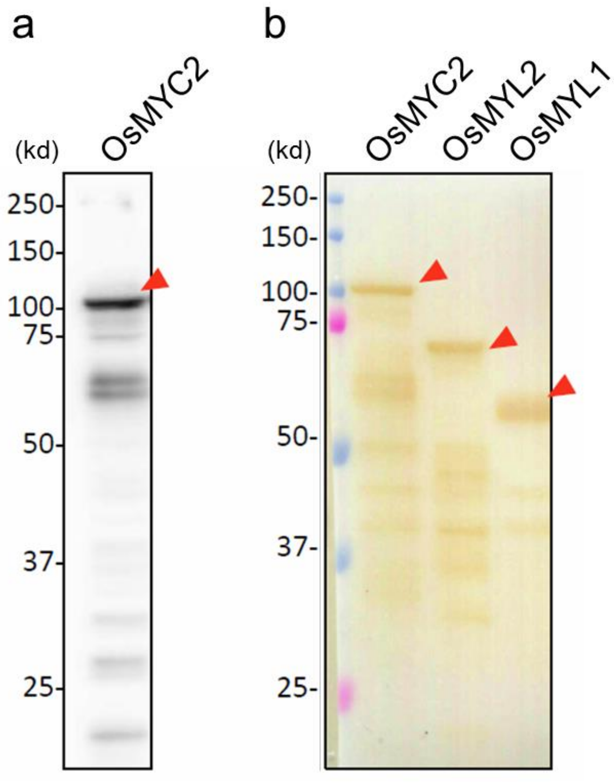

Supplementary Figure S4 **Recombinant proteins expressed using the wheat germ cell-free system.** Two micrograms of the crude protein was applied in each lane. Red arrows indicate the desired proteins. (**a**) Detection of biotinylated-OsMYC2 by using anti-Biotin HRP. (**b**) Detection of FLAG-tagged proteins by using anti-FLAG M2 HRP. A chemiluminescent reagent visualized the recombinant proteins without LAS detection.

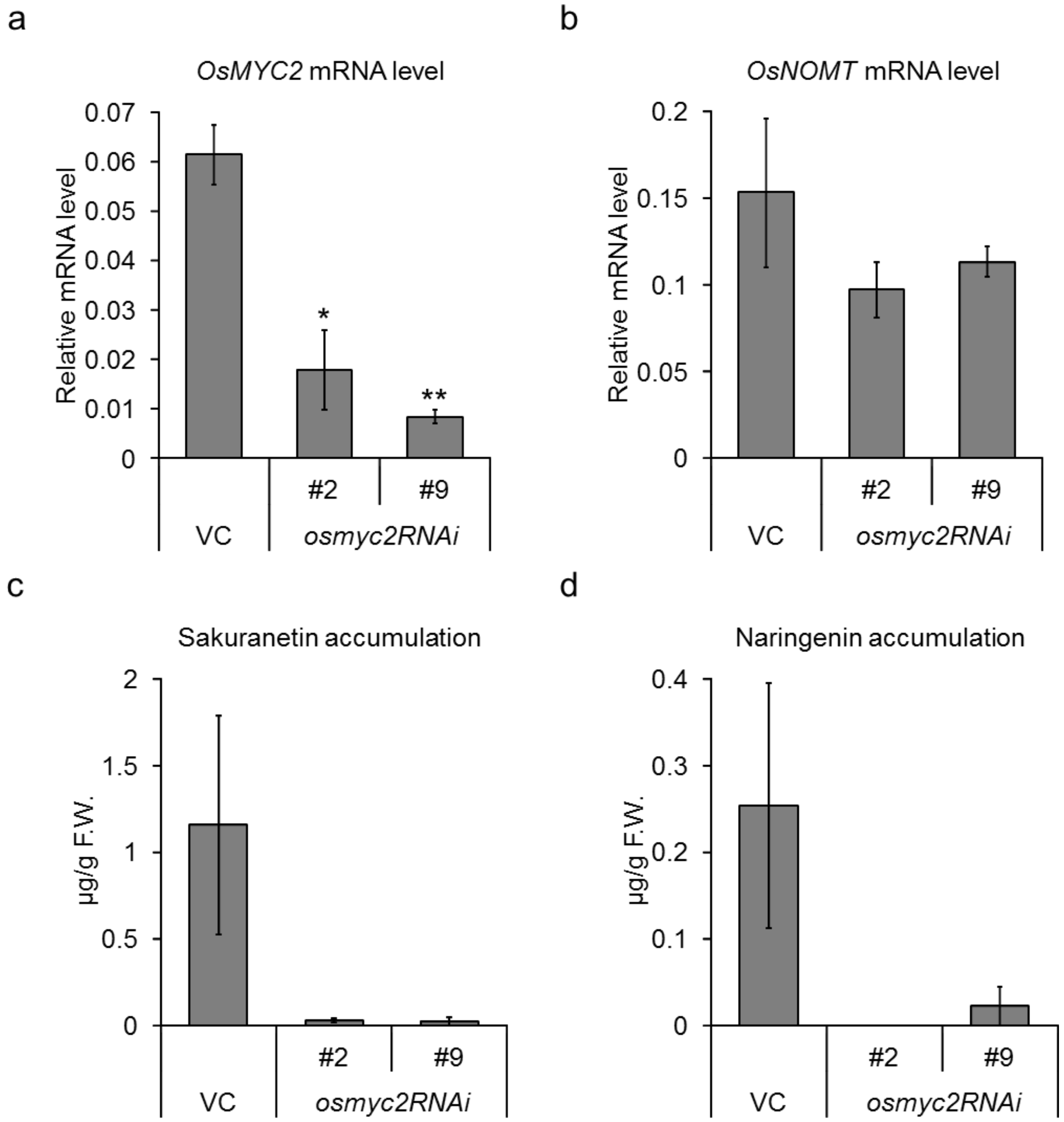

Supplementary Figure S5 **Gene expression and phytoalexin accumulation in the $CuCl_2$-treated *OsMYC2*-knockdown plants (*osmyc2RNAi*).** (**a, b**) Expression levels of *OsMYC2* (**a**) and *OsNOMT* (**b**). Total RNA from the rice leaves after 6 h of 500 µM $CuCl_2$ treatment was extracted. qRT-PCR analysis was performed using the cDNA samples synthesized from the total RNA. (**c, d**) Contents of sakuranetin (**c**) and naringenin (**d**) in the rice leaves. Data are presented as mean ± standard error values. (**a, b**) n = 3–4, (**c, d**) n = 4–6. *$P < 0.05$, **$P < 0.01$, compared to the VC data.

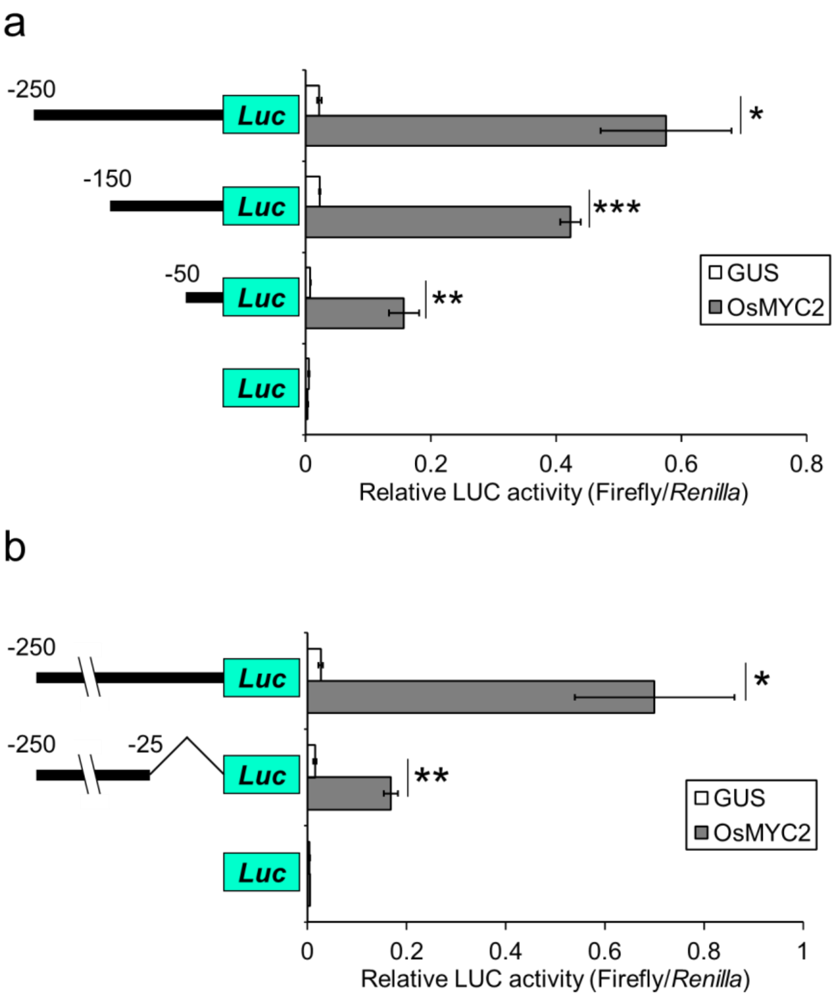

Supplementary Figure S6 **Deletion assay for the *OsNOMT* promoter.** 5′-truncated series (**a**) and 3′-truncated series (**b**) were used for the assay. Data are presented as FLUC/RLUC ± standard error. n = 4. *$P < 0.05$, **$P < 0.01$, ***$P < 0.001$.

Supplementary Table S1 **$CuCl_2$-induced expression of *OsNOMT* in transcriptome analysis.**

Gene ID	Gene name	WT $CuCl_2$ 2 h /WT water 2 h	Q value	WT $CuCl_2$ 6 h /WT water 6 h	Q value	WT $CuCl_2$ 24 h /WT water 24 h	Q value
Os12g0240900	OsNOMT	1.115	#	12.213	0.0038	17.002	0.0011

# indicates that fold change is ≤ 2 and/or that FDR is ≥ 0.05.

Supplementary Table S2 **JA-inducible transcriptional factors selected by transcriptome analysis using *cpm2*.**

Type	Gene ID	Gene name	Description	WT $CuCl_2$ 2 h /WT water 2 h	Q value	*cpm2* $CuCl_2$ 2 h/ WT $CuCl_2$ 2 h	Q value	WT $CuCl_2$ 6 h /WT water 6 h	Q value	*cpm2* $CuCl_2$ 6 h /WT $CuCl_2$ 6 h	Q value
HLH/bHLH	Os01g0108600		Basic helix-loop-helix dimerisation region bHLH domain containing protein. [Os01t0108600-01]	2.208	0.0031	0.081	0.0085	1.401	#	0.042	#
	Os01g0196300		Helix-loop-helix DNA-binding domain containing protein. [Os01t0196300-01]	65.337	0.0041	0.488	0.0204	130.220	#	2.781	#
	Os01g0705700	OsMYL1	Similar to Transcription factor ICE1 (Inducer of CBF expression 1) (Basic helix- loop-helix protein 116) (bHLH116) (AtbHLH116). [Os01t0705700-01]	70.452	0.0019	0.107	0.0015	49.453	0.0004	0.045	0.0054
	Os02g0120500		Helix-loop-helix DNA-binding domain containing protein. [Os02t0120500-02]	2.960	0.0337	0.327	0.0440	1.902	#	1.018	#
	Os02g0603600		Similar to Phytochrome-interacting factor 3 (Phytochrome-associated protein 3) (Basic helix-loop-helix protein 8) (bHLH8) (AtbHLH008). [Os02t0603600-01]	1.080	#	0.870	#	2.380	0.0080	0.401	0.0371
	Os03g0741100		Basic helix-loop-helix dimerisation region bHLH domain	38.999	0.0017	0.128	0.0183	22.264	#	0.229	#

			containing protein. [Os03t0741100-01]								
	Os04g0301500		Helix-loop-helix DNA-binding domain containing protein. [Os04t0301500-01]	631.858	0.0137	0.007	0.0066	58.968	#	0.016	#
	Os05g0163900		Helix-loop-helix DNA-binding domain containing protein. [Os05t0163900-01]	9.984	0.0137	0.092	0.0126	5.653	0.0009	0.344	0.0414
	Os07g0628500		Basic helix-loop-helix dimerisation region bHLH domain containing protein. [Os07t0628500-01]	1.398	#	0.483	#	3.197	0.0261	0.417	0.0210
	Os09g0501600		Similar to MYC1. [Os09t0501600-01]	1.901	#	0.417	#	6.826	0.0039	0.288	0.0035
	Os10g0376900		Helix-loop-helix DNA-binding domain containing protein. [Os10t0376900-01]	2.558	#	0.243	#	3.365	0.0013	0.139	0.0251
	Os10g0575000	OsMYC2	Similar to Transcription factor MYC7E (Fragment). [Os10t0575000-01]	3.177	0.0083	0.177	0.0103	2.355	0.0011	0.214	0.0179
MYB	Os01g0192300	JAdMYB3	Similar to R1MYB1 protein. [Os01t0192300-02]	15.686	0.0072	0.083	0.0396	9.418	0.0330	0.104	0.0117
	Os01g0702700	JAdMYB1	Similar to Transcription factor MYB86 (Myb-related protein 86) (AtMYB86) (Myb homolog 4) (AtMyb4). [Os01t0702700-01]	2.210	0.0101	0.032	0.0065	3.497	0.0027	0.010	0.0098
	Os02g0641300		Rep: ATMYB4-like - Oryza sativa subsp. japonica (Rice), complete [TC417408]	1.745	#	0.308	#	2.171	0.0063	0.319	0.0060

	Os05g0429900	JAdMYB4	Similar to MybHv5 (Fragment). [Os05t0429900-01]	10.548	0.0041	0.343	0.0016	11.496	0.0098	0.134	0.0009
	Os05g0442400		Similar to MybSt1. [Os05t0442400-01]	132.454	0.0128	0.014	0.0033	49.550	0.0306	0.046	0.0034
	Os05g0553400	OsMYB55	Similar to Myb-related transcription factor-like protein (MYB transcription factor). [Os05t0553400-01]	7.073	0.0025	0.409	0.0247	10.666	0.0025	0.194	0.0027
	Os08g0549000	JAdMYB5	Similar to MybHv5 (Fragment). [Os08t0549000-00]	4.410	0.0060	0.419	0.0123	5.257	0.0035	0.329	0.0191
	Os11g0684000	OsJAmyb	Similar to Transcription factor MYB21 (Myb-related protein 21) (AtMYB21) (Myb homolog 3) (AtMyb3). [Os11t0684000-01]	20.002	0.0008	0.126	0.0063	24.901	0.0004	0.070	0.0034
	Os12g0564100	JAdMYB2	Similar to R2R3MYB-domain protein (Fragment). [Os12t0564100-01]	4.645	0.0048	0.019	0.0037	2.439	0.0280	0.022	0.0203
bZIP	Os06g0662200		bZIP transcription factor, bZIP-1 domain containing protein. [Os06t0662200-01]	0.647	#	1.451	#	3.875	0.0070	0.079	0.0175
	Os08g0357300		Similar to BZIP DNA-binding protein. [Os08t0357300-01]	0.605	#	0.597	#	4.375	0.0007	0.195	0.0207
	Os12g0560900		Similar to BZIP protein. [Os12t0560900-01]	1.889	#	0.561	#	3.170	0.0027	0.443	0.0088
WRKY	Os01g0185900		Similar to WRKY 1 (Fragment). [Os01t0185900-01]	9.353	0.0009	0.413	0.0064	12.934	0.0008	0.466	0.0149
	Os01g0186000		Similar to WRKY transcription factor 10. [Os01t0186000-01]	24.393	0.0022	0.050	0.0159	26.186	0.0012	0.065	0.0057

	Os01g0626400	DNA-binding protein WRKY2-like (WRKY transcription factor 11) (WRKY16). [Os01t0626400-01]	5.141	0.0009	0.185	0.0092	9.051	0.0014	0.154	0.0027
	Os01g0714800	WRKY transcription factor 26. [Os01t0714800-01]	1478.633	0.0009	0.049	0.0075	1800.347	0.0003	0.363	0.0091
	Os01g0734000	Similar to WRKY DNA binding protein. [Os01t0734000-01]	4.291	0.0013	0.225	0.0108	3.804	0.0073	0.124	0.0066
	Os01g0750100	Similar to WRKY13 transcription factor. [Os01t0750100-03]	1.366	#	0.534	#	2.201	0.0033	0.320	0.0100
	Os01g0821600	WRKY transcription factor 48-like protein (WRKY transcription factor 21). [Os01t0821600-01]	58.586	0.0003	0.485	0.0072	35.706	#	0.679	#
	Os02g0770500	Similar to WRKY transcription factor 32. [Os02t0770500-00]	9.112	0.0009	0.196	0.0227	8.687	0.0020	0.149	0.0054
	Os05g0583000	WRKY transcription factor 34. [Os05t0583000-01]	1.790	#	0.194	#	2.318	0.0033	0.136	0.0058
	Os11g0117500	DNA-binding WRKY domain containing protein. [Os11t0117500-01]	398.079	0.0115	0.026	0.0015	731.906	0.0077	0.076	0.0109
	Os11g0490900	Similar to WRKY transcription factor 72. [Os11t0490900-01]	29.821	0.0011	0.321	0.0035	39.339	#	0.967	#
	Os12g0116700	Similar to WRKY transcription factor 64. [Os12t0116700-01]	345.859	0.0072	0.022	0.0021	582.052	0.0041	0.063	0.0041
NAC	Os01g0816100	Similar to NAC domain protein. [Os01t0816100-01]	140.804	0.0010	0.140	0.0047	199.857	0.0003	0.476	0.0231

	Os03g0777000		Similar to NAC-domain containing protein 19 (ANAC019) (ANAC) (Abscicic-acid-responsive NAC). [Os03t0777000-01]	10.184	0.0007	0.165	0.0068	14.617	#	0.910	#
	Os03g0815100		Similar to OsNAC6 protein. [Os03t0815100-01]	8.674	0.0012	0.489	0.0412	9.519	#	0.583	#
	Os08g0200600		Similar to NAC-domain containing protein 21/22 (ANAC021) (ANAC022). Splice isoform 2. [Os08t0200600-01]	1.512	#	0.830	#	5.387	0.0003	0.125	0.0045
	Os11g0126900		Similar to NAC domain transcription factor. [Os11t0126900-01]	2.607	0.0180	0.310	0.0066	1.163	#	0.219	#
(OsMYL2)	Os01g0235700	OsMYL2	Similar to BHLH transcription factor (Fragment).	1.027	#	1.004	#	1.156	#	0.971	#

# indicates that fold change is not sufficient for selection and/or that FDR is ≥ 0.05.

Supplementary Table S3 **Vectors used in this study.**

**Vector**	**Relevant characteristics**	**Reference**
pENTR/D-TOPO	Km^R, Gateway system entry vector	Invitrogen
pENTR-RLUC	Km^R, pENTR/D-TOPO containing *renilla luciferase* CDS	This study
pRL	Amp^R, CaMV 35S promoter-Renilla *LUC*	Promega
pUbi-RfA-Tnos	Amp^R, Cm^R, *ccdB*, pUCAP/Ubi-NT containing gateway cassette (RfA, invitorgen) cloned into *Bam*HI/*Sac*I sites after blunting	15
pUbi-RLUC	Amp^R, pUbi-RfA-Tnos containing *renilla luciferase* CDS	This study
pZErO2	Km^R, *ccdB*, cloning vector	Invitrogen
pZErO2-OsNOMT$_{1000}$	Km^R pZErO2 containing 5' 1-kb upstream region of *OsNOMT*	This study
pGL4-Tnos	Amp^R, Firefly *LUC-NOS* terminator	This study
pGL4-OsNOMT$_{1000}$	Amp^R, 5' 1-kb upstream region of *OsNOMT*- Firefly *LUC-NOS* terminator	This study
pENTR-OsMYC2	Km^R, pENTR/D-TOPO containing *OsMYC2* CDS	This study
pENTR-OsMYL1	Km^R, pENTR/D-TOPO containing *OsMYL1* CDS	This study
pENTR-OsMYL2	Km^R, pENTR/D-TOPO containing *OsMYL2* CDS	This study
pENTR-JAdMYB1	Km^R, pENTR/D-TOPO containing *JA-dependent (JAd) MYB1* CDS	This study
pENTR-JAdMYB2	Km^R, pENTR/D-TOPO containing *JAdMYB2* CDS	This study
pENTR-JAdMYB3	Km^R, pENTR/D-TOPO containing *JAdMYB3* CDS	This study
pENTR-JAdMYB4	Km^R, pENTR/D-TOPO containing *JAdMYB4* CDS	This study
pENTR-JAdMYB5	Km^R, pENTR/D-TOPO containing *JAdMYB5* CDS	This study
pENTR-OsJAmyb	Km^R, pENTR/D-TOPO containing *OsJAmyb* CDS	This study
pENTR-OsMYB55	Km^R, pENTR/D-TOPO containing *OsMYB55* CDS	This study
pUbi-GUS	Amp^R, pUbi_RfA containing 1.8 kb *GUS* flagment from pENTR-GUS	15
pUbi-OsMYC2	Amp^R, pUbi-RfA-Tnos containing *OsMYC2* CDS	This study
pUbi-OsMYL1	Amp^R, pUbi-RfA-Tnos containing *OsMYL1* CDS	This study
pUbi-OsMYL2	Amp^R, pUbi-RfA-Tnos containing *OsMYL2* CDS	This study
pUbi-JAdMYB1	Amp^R, pUbi-RfA-Tnos containing *JAdMYB1* CDS	This study
pUbi-JAdMYB2	Amp^R, pUbi-RfA-Tnos containing *JAdMYB2* CDS	This study
pUbi-JAdMYB3	Amp^R, pUbi-RfA-Tnos containing *JAdMYB3* CDS	This study
pUbi-JAdMYB4	Amp^R, pUbi-RfA-Tnos containing *JAdMYB4* CDS	This study
pUbi-JAdMYB5	Amp^R, pUbi-RfA-Tnos containing *JAdMYB5* CDS	This study
pUbi-OsJAmyb	Amp^R, pUbi-RfA-Tnos containing *OsJAmyb* CDS	This study
pUbi-OsMYB55	Amp^R, pUbi-RfA-Tnos containing *OsMYB55* CDS	This study
pENTR-OsMYC2 RNAi	Km^R, pENTR/D-TOPO containing 748-bp trigger region of *OsMYC2*	This study
pANDA	Hm^R, Km^R, Cm^R, *ccdB,* maize *ubiquitin* promoter-Gateway (reverse)-*gus* linker-Gateway-*NOS* terminator,	32
pANDA-OsMYC2	Hm^R, Km^R, pANDA containing 748-bp trigger region of *OsMYC2*	This study

pnYGW	Amp^R, Cm^R, *ccdB*, CaMV 35S promoter- nEYFP-Gateway-*NOS* terminator	34
pcYGW	Amp^R, Cm^R, *ccdB*, CaMV 35S promoter- cEYFP-Gateway-*NOS* terminator	34
pnYOsMYC2	Amp^R, pnYGW containing *OsMYC2* CDS	This study
pcYOsMYC2	Amp^R, pcYGW containing *OsMYC2* CDS	This study
pcYOsMYL1	Amp^R, pcYGW containing *OsMYL1* CDS	This study
pcYOsMYL2	Amp^R, pcYGW containing *OsMYL2* CDS	This study
pTH121R	Amp^R, CaMV 35S promoter-*DsRed1*-*NOS* terminator	17
pENTR-optOsMYC2	Km^R, pENTR/D-TOPO containing optimized *OsMYC2* CDS	This study
pEU-E01-6His-Bls-GW-STOP	Amp^R, Cm^R, *ccdB*, SP6 promoter-E01 translational enhancer-His tag-biotin ligation site-Gateway	This study
pEU-E01-FLAG-GW-STOP	Amp^R, Cm^R, *ccdB*, SP6 promoter-E01 translational enhancer-FLAG tag-Gateway	35
pEU-HisBls-optOsMYC2	Amp^R, pEU-E01-6His-Bls-GW containing optimized *OsMYC2* CDS	This study
pEU-FLAG-optOsMYC2	Amp^R, pEU-E01-FLAG-GW containing optimized *OsMYC2* CDS	This study
pEU-FLAG-OsMYL1	Amp^R, pEU-E01-FLAG-GW containing *OsMYL1* CDS	This study
pEU-FLAG-OsMYL2	Amp^R, pEU-E01-FLAG-GW containing *OsMYL2* CDS	This study
430T1.2	Amp^R, CaMV 35S promoter-GAL4 DNA binding domain-*NOS* terminator	36
p35S-GAL-OsMYC2	Amp^R, 430T1.2 containing *OsMYC2 CDS*	This study
p35S-GAL-OsMYL1	Amp^R, 430T1.2 containing *OsMYL1 CDS*	This study
p35S-GAL-OsMYL2	Amp^R, 430T1.2 containing *OsMYL2 CDS*	This study
GAL4-TATA-LUC-NOS	Amp^R, GAL4 *cis*-firefly *LUC-NOS* terminator	28

Supplementary Table S4 **Primers used for cloning.**

**Primer**	**Sequence**
RLUC Fw	5’-CACCATGACTTCGAAAGTTTATGATCCA-3’
RLUC Rv	5’-TTATTGTTCATTTTTGAGAACTCGCT-3’
OsNOMT$_{1000}$ Fw	5’-AAAGGTACCATAATTCTCCCATATATGTAGACAGTTTC-3’
OsNOMT$_{1000}$ Rv	5’-GGGGAGCTCGCTACTATACGGGGACACTG-3’
OsMYC2 Fw	5’-CACCATGAACCTTTGGACGGACGACAACGCGTC-3’
OsMYC2 Rv	5’-TTACCGGGCGGCGGTGCC-3’
OsMYL1 Fw	5’-CACCATGTCGTGGTCCGAGACG -3’
OsMYL1 Rv	5’-TCACGGGGAGGGAGTGGTG -3’
OsMYL2 Fw	5’-CACCATGGTGATGAAGATGGAGGC -3’
OsMYL2 Rv	5’-CTACCCTGAGTTCATCACGC -3’
JAdMYB1 Fw	5’-CACCATGGGACGGCACGCGTGCTC -3’
JAdMYB1 Rv	5’-TCATCGGAAGTACTCGAAGTTGAAGTCGCCGAC -3’
JAdMYB2 Fw	5’-CACCATGGCAGCATCGCAGAGGAG -3’
JAdMYB2 Rv	5’-TCATCGATTCCATATATAATTCGAATCAAATCGACATCG -3’
JAdMYB3 Fw	5’-CACCATGGCCAGGAAATGCTCCAGCTG -3’
JAdMYB3 Rv	5’-TCAAGTGACCCTGATTGTCCCCAAAAAAGG -3’
JAdMYB4 Fw	5’-CACCATGGGGAGGTCGCCGTGCTG -3’
JAdMYB4 Rv	5’-TCATATGTACTGGCCTTGTTCCAACGGCCTG -3’
JAdMYB5 Fw	5’-CACCATGGGGAGGTCGCCGTGCTG -3’
JAdMYB5 Rv	5’-TCATTTCATGGGGAGGCTTCTGAAGTCGAGC -3’
OsJAmyb Fw	5’-CACCATGGAGATGGTGCTGCAGAGGAC -3’
OsJAmyb Rv	5’-TTATTGCATCTTCCATATGTCCTCTAGACTCCATAGAG -3’
OsMYB55 Fw	5’-CACCATGGGGCGCGCGCCGTGCTG -3’
OsMYB55 Rv	5’-CTATGTCAGGGTGTTGCAGAGACCCTGTGGCTTGG -3’
OsMYC2 RNAi Fw	5’-CACCCTCCTACATCAACGAGCTCC-3’
OsMYC2 RNAi Fw	5’-ACAAGGACCAACATATCGCC-3’
pEU-bls-InFusion Fw	5’-AACGACATCTTCGAGGCCCAGAAGATCGAGTGGCACGAAACAAGTTTGTACAAAAAAGC-3’
pEU-bls-InFusion Rv	5’-CTCGAAGATGTCGTTCAGGCC**ATGATGGTGATGGTGATG**CATGATATCTTGGTGATGTAG-3’
optOsMYC2 Fw 1-	5’-CACCATGAACCTCTGGACCGATGATAACG-3’
optOsMYC2 Fw 671-	5’-GTGTTCTCGAGCTTGGATCTACCGATGTGATCTTCCAGAC-3’
optOsMYC2 Rv	5’-TCATCTAGCAGCAGTACCAGGTTC-3’
OsMYC2 GAL4 Fw	5’-GGAGGCCGAATTCCCGGGGATGAACCTTTGGACGGACGACAACGCGTC-3’
OsMYC2 GAL4 Rv	5’-GGTCGACGGATCCCCGGGTTACCGGGCGGCGGTGCC-3’
OsMYL1 GAL4 Fw	5’-GGAGGCCGAATTCCCGGGGATGTCGTGGTCCGAGACGGACG-3’
OsMYL1 GAL4 Rv	5’-GGTCGACGGATCCCCGGGTCACGGGGAGGGAGTGGTGAC-3’

OsMYL2 GAL4 Fw	5'-GGAGGCCGAATTCCCGGGGATGGTGATGAAGATGGAGGCTGATG-3'
OsMYL2 GAL4 Rv	5'-GGTCGACGGATCCCCGGGCTACCCTGAGTTCATCACGCGAG-3'

Restriction sites are underlined. Bls-tag epitope sequence is shaded. His tag sequence is shown in bold.

Supplementary Table S5 **Primers used for expression analyses.**

**Primer**	**Sequence**	**Target gene**
OsUBQ RT Fw	5’-TCCGAGAGATGGGTTTCATC -3’	*OsUBQ*
OsUBQ RT Fw	5’-GCCAAGATTGCCAAGAAGAC-3’	*OsUBQ*
OsMYC2 Fw	5’-AGCTCAACCAGCGCTTCTAC-3’	*OsMYC2*
OsMYC2 Rv	5’-GTCTCCTTGTCCGTCTCCAG-3’	*OsMYC2*
OsNOMT Fw	5’-AAGGTGTTCATGGAGAACTGGTA-3’	*OsNOMT*
OsNOMT Rv	5’-CTGGTTGAAGAGCGTGTTGGA-3’	*OsNOMT*

Supplementary Table S6 **Vectors referred to in the *Supplementary methods* section.**

**Vector**	**Relevant characteristics**	**Reference**
pUbi-RLUC	Amp^R, pUbi-RfA-Tnos containing *renilla luciferase* CDS	This study
pZErO2	Km^R, *ccdB*, cloning vector	Invitrogen
pZErO2-$OsNOMT_{250}$	Km^R pZErO2 containing 5' 250-bp upstream region of *OsNOMT*	This study
pZErO2-$OsNOMT_{150}$	Km^R pZErO2 containing 5' 150-bp upstream region of *OsNOMT*	This study
pZErO2-$OsNOMT_{50}$	Km^R pZErO2 containing 5' 50-bp upstream region of *OsNOMT*	This study
pZErO2-$OsNOMT_{250\text{-}26}$	Km^R pZErO2 containing 5' 250–26-bp upstream region of *OsNOMT*	This study
pGL4-Tnos	Amp^R, Firefly *LUC-NOS* terminator	This study
pGL4-$OsNOMT_{250}$	Amp^R, 5' 250-bp upstream region of *OsNOMT*- Firefly *LUC-NOS* terminator	This study
pGL4-$OsNOMT_{150}$	Amp^R, 5' 150-bp upstream region of *OsNOMT*- Firefly *LUC-NOS* terminator	This study
pGL4-$OsNOMT_{50}$	Amp^R, 5' 50-bp upstream region of *OsNOMT*- Firefly *LUC-NOS* terminator	This study
pGL4-$OsNOMT_{250\text{-}26}$	Amp^R, 5' 250–26-bp upstream region of *OsNOMT*- Firefly *LUC-NOS* terminator	This study
pUbi-GUS	Amp^R, pUbi_RfA containing 1.8 kb *GUS* flagment from pENTR-GUS	15
pUbi-OsMYC2	Amp^R, pUbi-RfA-Tnos containing *OsMYC2* CDS	This study

Supplementary Table S7 **Primers used for cloning and referred to in the *Supplementary methods* section.**

**Primer**	**Sequence**
OsNOMT$_{250}$ Fw	5’-AAACTCGAGTAGGAAGTTTAATGGACTTTTAGTAGATAATAG-3’
OsNOMT$_{250}$ Rv	5’-GGGAGATCTGCTACTATACGGGGACAC-3’
OsNOMT$_{150}$ Fw	5’-AAACTCGAGAACGTTGGAAAAAACCAACTACAC-3’
OsNOMT$_{150}$ Rv	5’-GGGAGATCTGCTACTATACGGGGACAC-3’
OsNOMT$_{50}$ Fw	5’-AAACTCGAGAGCTAGGTATCCTAGCTATATAAAC-3’
OsNOMT$_{50}$ Rv	5’-GGGAGATCTGCTACTATACGGGGACAC-3’
OsNOMT$_{250-26}$ Fw	5’-TAGGAAGTTTAATGGACTTTTAGTAGATAATAGATAAATAG-3’
OsNOMT$_{250-26}$ Rv	5’-GTTTATATAGCTAGGATACCTAGCTTATTGG-3’

Restriction sites are underlined.
